# Protecting the mental health of healthcare workers during the COVID-19 emergency

**DOI:** 10.1192/bji.2020.39

**Published:** 2020-06-18

**Authors:** Francesco Chirico, Gabriella Nucera, Nicola Magnavita

**Affiliations:** 1 Post-Graduate School of Occupational Medicine, Università Cattolica del Sacro Cuore, Rome, Italy. Email: medlavchirico@gmail.com; 2 Faculty of Nursing, University of Milan, Italy; 3 Post-Graduate School of Occupational Medicine, Università Cattolica del Sacro Cuore, Rome, Italy.

**Keywords:** Military psychiatry, post-traumatic stress disorder, psychosocial interventions, psychiatric nursing, epidemiology

## Abstract

Healthcare workers employed in the COVID-19 emergency are at high risk of stress, burnout and post-traumatic stress disorders. The most important occupational risk factors that employers should address include insufficient staff training and resources available, and lack of training and treatment protocols. In Italy, recent guidelines were released for providing all healthcare workers who are employed in this emergency with psychological support services based on coping strategies for managing stress. We suggest that preventive measures and a psychological intervention plan should be framed within the mandatory occupational health surveillance programme, and carried out by occupational physicians in cooperation with mental healthcare providers in the workplace.

Healthcare workers are on the front line of the battle against SARS-CoV-2 and COVID-19 disease and are paying the highest price for this global health emergency. As of 8 June 2020, there were 28 451 infected healthcare workers in Italy, and 167 doctors and 40 nurses had died.^1^ It is obvious that this public health emergency is creating stress in the general population, but healthcare workers who were already at high risk of stress, burnout and suicide before the COVID-19 pandemic began are experiencing even more stress.^2^ A recent study carried out in a tertiary infectious disease hospital in China revealed a high incidence of anxiety and stress disorders among front-line medical staff.^3^ In Italy, high levels of fear and anxiety have been reported by nurses and physicians, especially in the early stages of the current COVID-19 pandemic. Probably, as highlighted by Tsamakis et al in Greece, increasing knowledge about preventing and dealing with the disease, and the development of more specific procedural and treatment protocols, have contributed to improving the morale of healthcare workers dealing with the pandemic.^4^ Moreover, fear and anxiety may be caused by a lack of preparedness and stressful working conditions in hospitals, resulting from a lack of training and shortages of personal protective equipment (PPE). Furthermore, depression and burnout levels might be enhanced by excessive workloads and a shortage of healthcare personnel, as well as by the frustration and anger that result from watching people die without the support of their loved ones. In Italy, healthcare workers were emotionally struck by this crisis, and working in hospitals overwhelmed by COVID-19 cases, with a lack of effective treatments and shortages of equipment such as ventilators, has caused ethical dilemmas for healthcare workers.^5^

In Lombardy, the Italian region most severely affected by COVID-19, the healthcare service had been considered a benchmark in terms of quality and efficiency.^6^ Nevertheless, some medical doctors from Bergamo, during the weeks immediately preceding the peak of the epidemic, claimed to be working well below their normal standard of care. Patients had been waiting many hours for an intensive care bed, and older patients were not being resuscitated: they were dying alone without appropriate palliative care, while their family was notified over the phone, often by a well-intentioned but emotionally exhausted physician.^7^ Probably, the sudden spike of cases in this region made it impossible to meet the needs of many critically ill patients simultaneously. Therefore, many medical doctors had to deal with serious ethical dilemmas to which there were no right answers.

Moral injury is a form of psychological distress resulting from actions, or the lack of them, which infringe someone's moral or ethical code. In the UK, it has been recently described as a serious threat to the mental health of healthcare operators, who are facing intense feelings of shame, guilt or disgust due to the insufficient staff and resources available.^8^ Moral injury may be generated from moral dilemmas that front-line healthcare workers have to face during the COVID-19 pandemic and should be carefully considered as a serious threat to their mental health. Although moral injury is not comparable to a mental illness, it has been significantly associated with post-traumatic stress disorder, depression and suicidal ideation across a range of professions and could be specifically experienced by front-line key workers in this health emergency.

Despite the tremendous efforts that have been made by Italy in order to increase its critical care capacity, healthcare workers themselves are continuing to become severely sick. Consequently, they are obliged to avoid their families and friends, which increases the risk of post-traumatic stress disorder. This heavy emotional load may generate anxiety and depression, leading in the long term to burnout syndrome and, potentially, suicide. Italian newspapers recently reported suicide cases that involved two emergency nurses who tested positive for coronavirus and feared spreading it to their patients; other suicide cases among healthcare staff have been reported worldwide.

Protecting the mental health of healthcare professionals is a priority for policy makers. Moreover, physically and mentally exhausted workers are more likely to make mistakes at work, and could also be more susceptible to becoming infected. China has already developed a psychological intervention plan to support healthcare workers. Unfortunately, the plan encountered obstacles because medical staff were initially reluctant to participate in the group or in individual psychological interventions provided to them.^9^ However, these measures were adjusted to provide disease knowledge and protective measures for staff, as well as training to address issues such as the identification of and appropriate responses to psychological problems in patients with COVID-19. Other measures, including providing hospital security staff with detailed rules on the use and management of protective equipment, were successfully implemented. We believe these interventions are also needed in Italy and other countries.

In Italy, all healthcare and emergency workers providing services in healthcare settings or in the community (hospitals, emergency departments, prevention departments, epidemiological services, ambulance services and long-stay residential care homes, but also civil protection volunteers) are currently facing emotional overload and stress. Therefore, as proposed by the Italian Workers Compensation Authority and the Italian National Institute of Health, psychological support services are urgently needed and should be based on coping strategies for managing stress.^10^ We believe that it could be useful to implement psychological support resources within the framework of a mandatory occupational health surveillance programme, which is an effective and available instrument in healthcare settings. The psychological intervention plan should include two pillars: (a) providing healthcare workers with adequate information, training and PPE, in order to tackle the COVID-19 emergency; and (b) enhancing with psychological support the emotional skills of healthcare workers to deal with anxiety. Providing emotional support to patients and healthcare personnel through psychologists is also urgently needed. These measures should take into account the rules of military psychology during wartime scenarios, where application of an effective and accurate wartime triage is a team-based multidisciplinary activity and requires disaster preparedness and psychological skills. Moreover, spirituality care programmes and other approaches developed for end-of-life and palliative care could be useful. Finally, periodical medical examinations by occupational physicians in cooperation with mental healthcare providers for monitoring the mental health of healthcare workers should be developed immediately and maintained after the end of this emergency.
